# Molecular Biology of Cancer—Interplay of Malignant Cells with Emerging Therapies

**DOI:** 10.3390/ijms252313090

**Published:** 2024-12-05

**Authors:** Stergios Boussios, Matin Sheriff, Saak V. Ovsepian

**Affiliations:** 1 Faculty of Medicine, Health and Social Care, Canterbury Christ Church University, Canterbury CT1 1QU, UK; 2 Faculty of Life Sciences & Medicine, School of Cancer & Pharmaceutical Sciences, King’s College London, Strand, London WC2R 2LS, UK; 3 Kent Medway Medical School, University of Kent, Canterbury CT2 7LX, UK; 4 AELIA Organization, 9th Km Thessaloniki–Thermi, 57001 Thessaloniki, Greece; 5 Department of Medical Oncology, Medway NHS Foundation Trust, Gillingham ME7 5NY, UK; 6 Department of Urology, Medway NHS Foundation Trust, Gillingham ME7 5NY, UK; matin.sheriff@nhs.net; 7 Faculty of Engineering and Science, University of Greenwich London, Chatham Maritime, Gillingham ME4 4AG, UK; s.v.ovsepian@greenwich.ac.uk; 8 Faculty of Medicine, Tbilisi State University, Tbilisi 0179, Georgia

## Discussion

Cancer is currently one of the leading causes of death worldwide, and according to data from the World Health Organization reported in 2020, it ranks as the second leading cause of death globally, accounting for 10 million fatalities [1]. Over the past century, our understanding of cancer biology has advanced remarkably. This progress has been especially accelerated in recent decades due to technological and conceptual breakthroughs in various fields, such as next-generation sequencing, omics sciences, high-resolution microscopy, molecular immunology, flow cytometry, single-cell analysis and sequencing, new cell culture methods, and the development of animal models, among others [2,3,4,5]. Despite these advances, many questions remain unanswered, and numerous challenges persist in addressing this disease, so oncological research remains essential. Cancer is strongly linked to genetic factors, with oncogenesis playing a crucial role in the initial stages of tumor formation [6]. Most common cancers arise from acquired mutations in somatic cells, while specific germline mutations are responsible for rare hereditary cancer syndromes [7]. Among cancer-related genes, oncogenes become activated, exhibiting a dominant phenotype, while tumor suppressor genes are inactivated, showing a recessive phenotype. The accurate diagnosis, prognosis, and prediction of cancer patients’ responses to treatment are crucial for ensuring the most effective engagement, minimizing harmful side effects, and targeting therapies to specific cancer mechanisms [8]. To this end, the molecular biology of cancer has become increasingly vital in oncology. This Special Issue, entitled “Molecular Biology of Cancer—Implications for Diagnosis and Treatment: 2nd Edition” includes twelve contributions, consisting of nine original articles and three reviews, offering new insights into cancer biology, molecular genetics, and innovative therapeutic strategies.

Immune checkpoint blockade therapies treat cancer by lifting inhibitory signals and activating the host’s immune system. One of the breakthroughs in immunotherapy has been the successful use of treatments that block the Programmed Cell Death Protein 1 (PD-1)/Programmed Cell Death Ligand 1 (PD-L1) pathway, effectively treating various cancers [9]. However, a significant fraction of tumors remain resistant to these therapies [10], and interest has been increasing in exploring immune pathways to identify new therapeutic targets. One such target is the human endogenous retrovirus-H Long repeat-associating 2 (HHLA2), a member of the B7 family that was first identified in 1999 [11]. HHLA2 is an unconventional checkpoint molecule within the B7 family, structurally unique that is not expressed in mice or rats. It is consistently expressed in human antigen-presenting cells (APCs) and normal tissues, and its expression is elevated in various cancers. HHLA2 interacts with the co-stimulatory Transmembrane and Immunoglobulin Domain Containing 2 (TMIGD2) and the co-inhibitory Killer Cell Immunoglobulin-Like Receptor, Three Ig Domains, and Long Cytoplasmic Tail 3 (KIR3DL3) [12]. TMIGD2 is found in naïve T cells, memory T lymphocytes, tissue-resident T cells, natural killer (NK) cells, plasmacytoid dendritic cells, and innate lymphoid cells. The HHLA2 and TMIGD2 interaction activates the proliferation and differentiation of T cells and enhance cellular cytotoxicity effect of NK cells [13]. Conversely, KIR3DL3 inhibits NK cell function and mediates HHLA2 tumor resistance against NK cells. As T cells become activated, TMIGD2 expression decreases, while KIR3DL3 expression increases, enhancing HHLA2′s co-inhibitory functions. Tumors may use the KIR3DL3-HHLA2 pathway to evade immune surveillance, making this pathway a promising target for new immunotherapy approaches [11]. The prognostic value of HHLA2 in various cancers is still uncertain.

Kula et al. performed a systematic review and meta-analysis of existing studies, encompassing 18 reports and a total of 2880 patients with solid tumors (Contribution 1). The meta-analysis showed that elevated HHLA2 expression is linked to a poor prognosis. Elevated HHLA2 levels were specifically identified as a risk factor for overall survival (OS) (HR = 1.58, 95% CI: 1.23–2.03) and relapse-free survival (RFS) (HR = 1.95, 95% CI: 1.38–2.77). Furthermore, the analysis revealed that patients with high HHLA2 expression had poorer disease-free survival (DFS) compared to those with low expression. A subgroup analysis of gastrointestinal cancers showed that patients with high HHLA2 expression had shorter OS (random-effects model, HR = 1.88, 95% CI: 1.55–2.28). However, the meta-analysis did not find a significant association between high HHLA2 expression and shorter progression-free survival (PFS) (HR = 1.07, 95% CI: 0.43–2.63) or disease-specific survival (DSS) (HR = 1.52, 95% CI: 0.88–2.62). Moreover, high HHLA2 expression was linked to poor OS regardless of its location within the tumor. The results of the meta-analysis of Kula et al. revealed significant heterogeneity among the included studies. A key factor contributing to this high heterogeneity is the use of different cut-off values for HHLA2 levels to distinguish between high and low expression in different cohorts. Most studies assessed HHLA2 expression using H-scores, which are calculated by multiplying the percentage of tumor cells expressing HHLA2 by the intensity of the staining. However, the use of varying methods to categorize tumors into high- and low-expression groups makes it difficult to compare studies and complicates the meta-analysis results. Further research is essential to fully uncover the prognostic significance of HHLA2 expression in patients with solid tumors.

Liquid biopsy, which involves analyzing biomarkers that tumors release into body fluids, is an emerging area in translational cancer research. It allows for dynamic patient monitoring and a more personalized approach to medicine [14]. Within this context, there has been a significant surge in interest in extracellular vesicles (EVs). These small, membrane-encapsulated particles are released by various cell types and can be found in most biological fluids. EVs play a crucial role in intercellular signaling under both physiological and pathological conditions, including cancer, because they carry a variety of bioactive molecules derived from their cells of origin, such as microRNAs (miRNAs) and long non-coding RNA (lncRNA) [15]. Compared to free circulating miRNAs and lncRNAs, those contained within EVs are better protected by the lipid bilayer, which enhances their stability and half-life. This makes EV-derived miRNAs and lncRNAs particularly attractive for research and quantification in biological fluids, offering potential diagnostic, prognostic, and therapeutic applications in oncology.

Giordano et al. performed next-generation sequencing (NGS) followed by quantitative reverse transcription polymerase chain reaction (qRT-PCR) to investigate the differences in circulating EV-miRNA profiles between breast cancer patients and healthy controls (Contribution 2). They found a significant reduction in miRNA-27a expression within EVs in both the breast cancer screening and validation cohorts. Receiver operating characteristic (ROC) analyses indicated that circulating EV-derived miRNA-27a effectively distinguished breast cancer patients from healthy individuals, achieving a favorable area under the curve (AUC) value, which suggests its potential utility in breast cancer diagnosis. Additionally, the expression of miRNA-27a did not correlate with human epidermal growth factor receptor 2 (HER2) status or tumor grade, but it was associated with hormone receptor status and Ki-67 levels. The authors could not reveal a difference in serum miRNA-27a and miRNA-128 levels between patients and controls. However, there was a distinct discrepancy between the miRNA profiles of EVs and serum, indicating that purified serum-EV miRNA-27a and miRNA-128 may offer higher diagnostic accuracy for breast cancer compared to bulk serum miRNAs. Table 1 summarizes the results of 11 studies regarding biofluid type and miRNA levels in the relevant population [16,17,18,19,20,21,22,23,24,25,26]. Larger prospective studies are needed to more accurately assess the potential diagnostic value of cell-free miRNA-27a and/or miRNA-128 (whether circulating or EV-derived) as sensitive and specific non-invasive molecular biomarkers.

Mammary gland cancer usually arises in the epithelial cells of the ducts, where the accumulation of mutations can lead to cellular damage [27]. The tissue repair process that follows this damage triggers an inflammatory response, causing both quantitative and qualitative shifts in the immune cell population at the injury site. Chronic inflammation, in turn, can promote additional cell mutations and proliferation, often creating a microenvironment that supports cancer development [28]. The interleukin 1 (IL-1) family, known for its diverse effects on inflammation, includes numerous ligands and receptors, with IL-1α and IL-1β being the two primary agonists [29,30]. Interleukin 1 receptor type 1 (IL1R1) is the main receptor for both ligands and forms a tertiary complex with the IL-1 receptor accessory protein (IL1RaP, also known as IL1R3), which facilitates positive signaling and recruits downstream proteins [31,32]. However, IL1R1 also has an antagonistic ligand (IL1RN, also known as IL1RA), which can be either overexpressed or downregulated in different types of cancer [31,32,33,34,35]. Interestingly, high IL1RN expression is associated with both better [32,36,37,38] and worse cancer prognoses [37,39], depending on the context. The Interleukin 6 (IL-6)-like family consists of proteins with similar structural and functional characteristics. The IL-6 signal transducer (IL6ST, also known by its gene name) is a transmembrane receptor that functions as a signal transducer for all cytokines within this family [40]. Its functions are closely linked to many of the key hallmarks of cancer development and progression [41,42,43].

Koning et al. (Contribution 3) investigated the expression of inflammation-related genes, including IL1R1, interleukin 1 receptor antagonist (IL1RN), ILRaP, IL6ST, C-X-C motif chemokine ligand 3 (CXCL3), C-X-C motif chemokine ligand 5 (CXCL5), and C-X-C motif chemokine ligand 6 (CXCL6), using the previously established Alpha Model of estrogen and radiation-induced breast cancer [44]. They compared the gene expression in the Alpha Model with data from online breast cancer patient databases, focusing on the expression of these genes in innate immune system cells, various breast cancer subtypes, and estrogen receptor (ER) alpha. Additionally, the study results revealed variations in the expression of inflammatory genes at different stages of breast cell transformation in the Alpha model. To compare the gene expression between healthy individuals and breast cancer patients, the TIMER2.0 online database platform was used. The findings revealed that IL1RN expression was higher in tumor tissue compared to adjacent normal tissue. Conversely, IL1R1, ILRaP, IL6ST, CXCL3, CXCL5, and CXCL6 genes were more highly expressed in adjacent normal tissue than in breast tumor tissue. IL1R1 gene expression was significantly associated with an increased risk (Z-score, *p* < 0.05) for patients with Luminal A subtype breast invasive carcinoma, whereas higher IL1RaP gene expression was linked to an increased risk in Luminal B patients. However, based on the Cox proportional hazard model, the expression levels of the IL1RN, IL6ST, CXCL3, CXCL5, and CXCL6 genes were not significantly associated with clinical survival in breast cancer patients. Luminal A patients with low IL1R1 expression had better survival than those with high IL1R1 expression, who showed no survival beyond 150 months. For Luminal B patients, high IL1RaP expression was linked to lower survival rates compared to low IL1RaP expression. This survival disparity was observed between 30 and 130 months, after which both high and low IL1RaP expression patients had the same cumulative survival rate of 0.2. Patients with high IL6ST expression levels were found to have a positive ER status. However, those with elevated IL1RaP, CXCL3, CXCL5, and CXCL6 expression levels were associated with a negative ER status. The study by Koning et al. highlighted the crucial role of both the IL-1 gene family and chemokines in the development and progression of breast cancer. In terms of the current clinic practice, the European Society for Medical Oncology (ESMO) guidelines for early breast cancer recommend using immunohistochemistry to evaluate classical molecular markers, including estrogen receptors (ER), progesterone receptors (PR), HER2, and Ki-67, as well as Sanger sequencing or NGS to identify specific gene alterations [45]. Germline *Breast Cancer gene 1* and *2 (BRCA1* and *BRCA2)* mutation testing is recommended for patients with a family history of breast cancer, a personal history of ovarian cancer, breast cancer diagnosed before age 50, triple-negative breast cancer diagnosed before age 60, and male patients [46]. For metastatic breast cancer, the guidelines suggest the classical biomarkers along with germline *BRCA* mutation testing for patients with triple-negative breast cancer or ER-positive, HER2-negative breast cancer, while *phosphatidylinositol-4,5-bisphosphate 3-kinase catalytic subunit alpha (PIK3CA)* mutation testing is optional in both cases. The genomic profiling of additional tumor tissue or circulating tumor DNA (ctDNA) testing is suggested only if the results would influence the treatment plan or make the patient eligible for clinical trials (Table 2).

Colorectal cancer (CRC) ranks as the third most frequently diagnosed cancer, after breast and lung cancer, and the second most common cause of cancer-related deaths worldwide [1]. Survival rates for CRC differ markedly by stage [47]. Patients with stage III CRC have a 5-year survival rate of 72%, whereas those with stage IV disease face a significantly lower survival rate of 13% [48]. For stage III colon cancer, the standard treatment involves surgery followed by adjuvant chemotherapy, whereas the benefit of adjuvant chemotherapy for resected stage II disease is less well-established [49,50]. Identifying and validating biomarkers for CRC is essential for selecting high-risk patients who may benefit from adjuvant chemotherapy [51]. Several biomarkers are currently used to conduct a comprehensive assessment of CRC, including carcinoembryonic antigen (CEA), mismatch repair deficiency (MMR), microsatellite instability (MSI), v-raf murine sarcoma viral oncogene homolog B1 (BRAF) mutations, reticular activating system (RAS) mutations, and caudal-type homeobox transcription factor 2 (CDX2) [52]. Numerous studies have shown that the expression of various miRNAs in CRC patients differs significantly from that in the general population. These differences can serve as valuable biomarkers for the early diagnosis and prognosis of CRC [53]. As new non-invasive biomarkers, these miRNAs demonstrate high accuracy and specificity in early CRC detection, marking a significant advancement in non-invasive diagnostic techniques. Table 3 presents miRNAs used as biomarkers for the early diagnosis of colorectal cancer.

The CDX2 protein family, which is part of the ParaHox gene cluster, consists of three members—CDX1, CDX2, and CDX4 [65,66]. A deficiency in CDX2 results in compromised barrier function and enterocyte atrophy, and it is thought that the loss of CDX2 is primarily due to epigenetic changes rather than genetic mutations [67]. CDX2 downregulation or loss has been observed in a subset of CRC, and the absence of CDX2 expression is associated with more aggressive features, including advanced stage, poor differentiation, vascular invasion, and BRAF mutation, all of which can contribute to lower survival rates [68,69,70]. Evidence in the literature indicates that patients with CDX2-negative tumors typically have lower survival rates than those with CDX2-positive tumors. Additionally, the loss of CDX2 expression may help identify a subset of stage II colon cancer patients at increased risk for disease recurrence who could benefit from adjuvant chemotherapy [71]. These findings highlight the potential of CDX2 as both a prognostic biomarker, offering insights into disease progression or outcomes regardless of treatment, and a predictive biomarker, which provides information on the likelihood of response to specific therapies. The reported incidence of CDX2-negative cases varies widely, ranging from 5% to 30% across different studies, as illustrated in Table 4 [71,72,73,74,75,76,77,78,79].

In a retrospective study, Chan et al. evaluated a cohort of patients with stage I to IV CRC for CDX2 staining and analyzed its correlation with patient demographics, clinicopathologic features, and cancer outcomes, including OS, DFS, and RFS (Contribution 4). Additionally, they analyzed these parameters separately for colon and rectal cancer patients. Low CDX2 expression was significantly associated with adverse prognostic features, including poor tumor differentiation, lymphovascular or perineural invasion, and reduced OS and DFS in both colon and rectal cancers. In addition to assessing the prognostic value of CDX2 expression, Chan et al. explored its potential as a predictive biomarker. The study showed a trend toward improved survival with adjuvant chemotherapy in patients with low CDX2 expression, although this trend did not achieve statistical significance (*p* = 0.113). In stage III colon cancer, adjuvant chemotherapy was associated with a statistically significant improvement in OS for both high (HR 0.19 [CI 0.11–0.32]), *p* < 0.0001)- and low (HR 0.05, [CI 0.01–0.23], *p* < 0.001)-CDX2 expression tumors. However, this result is less clinically impactful because most stage III colon cancer patients are typically recommended adjuvant chemotherapy irrespective of CDX2 expression. The study by Chan et al. also investigated the role of CDX2 in predicting risk for rectal cancer, an area with limited literature compared to studies combining colon and rectal cancers. The lack of significant differences in survival outcomes between patients with high and low CDX2 expression who received adjuvant chemotherapy for rectal cancer suggests that CDX2 is unlikely to predict chemotherapy response in this group. Indeed, OS improved regardless of whether their CDX2 expression was high (*p* = 0.017) or low (*p* < 0.0001). Furthermore, the impact of adjuvant therapy on observed outcomes is uncertain, especially since some patients had received pre-operative treatment. Additionally, CDX2 expression did not correlate with treatment outcomes in the rectal cancer cohort undergoing neoadjuvant therapy (*p* = 0.825). The study also revealed a significant increase in the percentage of cases with CDX2 expression loss as cancer stage advanced, with rates of 3.5% in stage I, 5.0% in stage II, 10.7% in stage III, and 12.1% in stage IV (*p* = 0.015). Two clinicopathological features significantly associated with CDX2 expression were tumor differentiation (*p* < 0.001) and the presence of lymphovascular or perineural invasion (*p* = 0.002). Regarding tumor sidedness, low CDX2 expression was more common in right-sided tumors (11.7%) compared to left-sided tumors (6.0%), which was statistically significant. Similarly, there was a higher proportion of low CDX2 expression in colon tumors (9.2%) versus rectal tumors (5.7%), although this difference was not statistically significant.

Apocrine gland anal sac adenocarcinoma (AGASACA) is a relatively uncommon tumor in dogs, accounting for approximately 17% of perianal malignancies [80,81]. These neoplasms exhibit variable local aggressiveness and a high potential for metastasis [80,81,82]. Surgical excision is the gold standard, whereas additional treatment options include chemotherapy, radiotherapy, electrochemotherapy, molecular targeted therapy, and cyclooxygenase-2 inhibitors [81,83,84,85]. HER2 is a membrane protein belonging to the epidermal growth factor receptor (EGFR/HER) family, normally expressed in various tissues. When overexpressed, the HER2 receptor initiates proliferative signals and disrupts cellular control mechanisms, contributing to tumorigenesis [86,87,88]. HER2 overexpression has been observed in several types of neoplasms and its potential as a therapeutic target has been explored through the use of anti-HER2 monoclonal antibodies [89,90,91,92]. Ki67, a nuclear protein associated with cell proliferation, is present in cells undergoing active division and is either minimal or absent in quiescent cells [93,94]. Its expression is detected in all proliferating cells, both normal and neoplastic, and is characterized by a short half-life of approximately one hour [80,94,95]. Ki67 is widely used as a key marker of cell proliferation, serving as a prognostic indicator of the proliferative index across various tumor types.

Paiva et al. investigated the expression of HER2 and Ki67 in cases of canine AGASACA, focusing on their presence and clinical significance (Contribution 5). Immunohistochemistry (IHC) showed that 45% of the neoplastic cases were positive for HER2 staining, while the control group exhibited 100% negative HER2 staining. Several studies have explored tumor size as a prognostic factor in AGASACA cases, using various cutoff values [82,96,97]. The most widely accepted size criteria come from Polton and Brearley’s 2007 staging model, where measurements are conducted through clinical evaluation with a caliper [98]. However, in the study by Paiva et al., tumor size was measured directly on formalin-fixed tissue. Evaluating the primary tumor samples including T1 (largest tumor diameter smaller than 2.5 cm) and T2 (largest tumor diameter larger than 2.5 cm), positive HER2 staining was observed in 45% of the cases, all of which had a score of 2+, with no cases scoring 3+. A statistical analysis showed no correlation between HER2 expression and tumor size. This suggests that HER2 expression is present even in smaller tumors diagnosed at early stages, indicating that these cases might also benefit from targeted therapies. HER2 expression was also detected in metastatic lymph node samples, with no statistically significant difference compared to primary tumors, making it difficult to assess its effectiveness as a prognostic marker. Ki67 expression was measured at 25% across all groups, except for the control group, which had a median expression of 8%. Other studies have reported higher levels, with a median of 34.33% and a mean of 34.58% (ranging from 19.6% to 55.98%), as well as lower levels, with a median of 7.75% (ranging from 0% to 54%) [99,100]. Overall, both HER2 and Ki67 show distinct expression and represent promising therapeutic targets for the development of new anticancer therapies.

The heterodimerization of HER2 with other members of the HER family, or its ligand-independent homodimerization, results in the autophosphorylation of the cytoplasmic domain. HER2 overexpression is observed in approximately 20% of breast cancers and 20% of gastric cancers and is linked to higher recurrence rates and reduced OS [101,102]. Trastuzumab, an anti-HER2 monoclonal antibody, has shown anti-proliferative activity in vitro and significant antitumor effects in vivo [103,104]. This has resulted in its approval by the U.S. Food and Drug Administration (FDA) for the treatment of HER2-positive breast cancer [105]. Trastuzumab is given to patients with tumors that overexpress HER2, which is characterized by solid, complete membranous staining of more than 10% of cells in IHC (IHC 3+) and/or amplification detected by in situ hybridization (ISH) [106]. Additionally, trastuzumab–deruxtecan (T-DXd), an antibody-drug conjugate (ADC) based on trastuzumab, has also received FDA approval [107]. T-DXd demonstrates greater efficacy not only in HER2-positive breast cancers but also in HER2-low (IHC 1+ or IHC 2+/ISH non-amplified) advanced breast cancers and HER2-mutant lung cancers [108,109,110,111,112]. Since half of all breast cancers are classified as HER2-low, a substantial number of patients are likely to benefit from T-DXd therapy [113]. Cardiotoxicity is a common adverse effect associated with anti-HER2 monoclonal antibodies and ADCs, necessitating routine cardiac monitoring for patients [114]. Research teams have developed CasMabs targeting HER2 (H2Mab-250), podocalyxin (PcMab-6), and podoplanin (LpMab-2 and LpMab-23), and evaluated their reactivity against both cancer and normal cells using flow cytometry and IHC [115,116,117]. H2Mab-250 exhibits a strong in vivo antitumor effect, despite its lower in vitro reactivity and affinity compared to trastuzumab [115,116]. The underlying reasons for this discrepancy have not yet been clarified.

Suzuki et al. compared the antibody-dependent cellular cytotoxicity (ADCC) and complement-dependent cytotoxicity (CDC) of H2Mab-250 and trastuzumab (Contribution 6). They found that H2Mab-250 demonstrated superior CDC activity against breast cancer and HER2-overexpressing cells compared to trastuzumab. Moreover, both H2Mab-250-hG1 and H2Mab-250-mG2a showed comparable antitumor effects to those of trastuzumab’s corresponding isotypes. These findings suggest that the enhanced CDC activity of H2Mab-250 may offset its lower ADCC activity, contributing to its overall antitumor efficacy. Chimeric antigen receptor (CAR)-T cell therapy is making significant strides as an anticancer treatment, but designing the optimal CAR remains a challenge [118]. In solid tumors, immune checkpoints such as PD-1, T cell immunoglobulin and mucin-domain-containing 3 (TIM-3), and CTLA-4 are often overexpressed, leading to CAR-T cell exhaustion. The tumor microenvironment (TME) comprises immunosuppressive cells, including regulatory T cells (Tregs), myeloid-derived suppressor cells (MDSCs), and M2 macrophages. These cells release cytokines such as transforming growth factor-beta (TGFβ) and interleukin 10 (IL-10), which diminish the effectiveness of CAR-T cells in targeting tumors [119]. To tackle this issue, strategies include combining CAR-T cells with immune checkpoint inhibitors, like PD-1 inhibitors, or genetically modifying CAR-T cells to enhance their immune response and resistance to inhibitory factors, thereby improving their anti-tumor efficacy [120]. Table 5 summarizes several clinical trials exploring the combination of CAR-T cells with immune checkpoint blockades.

H2Mab-250 is currently being developed for CAR-T cell therapy and is undergoing evaluation in a phase I trial for HER2-positive advanced solid tumors in the U.S. (NCT06241456). Research indicates that resistance to CDC can arise from the expression of complement regulators in tumor cells, facilitating their evasion of host immune responses [121]. The upregulation of these regulators, such as CD46, CD55, and CD59, has been shown to inhibit CDC by blocking terminal complement activation and preventing the formation of the membrane attack complex [122,123]. Future studies should investigate the dual targeting of HER2 by H2Mab-250 and complement regulators in in vitro models.

Bladder cancer is the tenth most commonly diagnosed cancer globally and represents a major cause of morbidity and mortality [124]. Urothelial carcinoma is the most prevalent histologic type; nevertheless, squamous cell carcinoma is more frequently observed in regions of Africa where schistosomiasis is widespread [125]. Non-muscle-invasive bladder cancer (NMIBC) comprises around 75% of bladder cancer cases and is characterized by a high recurrence rate, which can be mitigated with *Mycobacterium bovis Bacillus Calmette–Guérin* (BCG) immunotherapy [126]. However, BCG immunotherapy fails in 30–50% of patients, and the frequent monitoring required contributes to high disease management costs [127,128,129]. Research into the mechanisms underlying BCG’s action has led to the investigation of single-nucleotide polymorphisms (SNPs) in genes related to cytokine production, DNA repair pathways, and reactive oxygen species production for their potential to predict responses to BCG therapy [130,131,132,133,134]. GSTT2 is a member of the glutathione-S-transferase (GST) family of proteins, which detoxifies both xenobiotics and endobiotics by conjugating them with glutathione (GSH). The pseudogene GSTT2B is a duplicate of GSTT2 in a head-to-head arrangement. It is commonly lost in humans, leading to a significant reduction in GSTT2 expression [135]. Higher GSTT2 expression has been linked to a lower risk of colon cancer, esophageal squamous cell carcinoma, and Barrett’s esophagus [136,137,138].

Rahmat et al. found that GSTT2 expression plays a crucial role in the intracellular survival of BCG in host cells (Contribution 7). It has also been shown to act on secondary lipid peroxidation products and organic hydroperoxides [139]. GSTT2 may facilitate BCG elimination by regulating intracellular levels of these secondary lipid peroxidation products. When bladder cancer cells internalize live BCG, there is an increase in cellular reactive oxygen species (ROS) levels and lipid peroxidation, while lyophilized BCG induces the opposite effect [140]. Rahmat et al. also observed that GSTT2 expression influences ROS generation induced by BCG. In vitro, overexpression of GSTT2 resulted in increased net ROS levels following treatment with lyophilized BCG and decreased survival of intracellular live BCG. This suggests that after exposure to live BCG, the overexpression of GSTT2 might deplete the intracellular supply of GSH, which is necessary to counteract the effects of lipid peroxidation. The depletion of GSH would lead to elevated net ROS levels, thereby contributing to the destruction of BCG particles via ROS. In contrast, in GSTT2-silenced cells, GSTT2 activity does not deplete intracellular GSH following live BCG treatment, resulting in lower net ROS levels and increased BCG survival. Overall, the study by Rahmat et al. suggests that tailoring patient responses based on genotype may be feasible. Genotyping could also help reduce the frequency of post-BCG therapy surveillance for some patients.

Uterine myomas are the most common benign tumors of the female reproductive system [141]. Also known as fibroids, they develop from smooth muscle cells and affect more than 70% of women of reproductive age [142]. The development and growth of myomas are influenced by various factors, such as steroid hormones, multiple cytokines, growth factors, the extracellular matrix (ECM), microRNA, genetic predispositions, stem cells, and vitamin D deficiency [143,144,145,146]. Angiogenesis is a complex process that involves stimulatory factors, components of the ECM, and various cell types, and among the factors that stimulate angiogenesis are angiopoietins. Angiopoietin 1 (ANG1), one of the three well-known angiopoietins, is encoded by the *ANGPT1* gene located on chromosome 8q23.1 and is expressed in smooth muscle cells, fibroblasts, and pericytes. The synthesis of ANG1 is regulated by the hypoxia-inducible factor alpha (HIF-1α) and is associated with calcium ions [147]. ANG1 has a localized effect on the ECM, with cell signaling mediated by protein kinases such as Mitogen-activated Protein Kinase (MAPK) and Focal Adhesion Kinase (FAK) [143,148]. The angiogenic functions of ANG1 are influenced by calcium signaling pathways that depend on Ca^2+^ [149]. Calcium homeostasis is regulated by the calcium-sensing receptor (CaSR), which is primarily expressed in the parathyroid glands, kidneys, and brain and plays a role in numerous biological processes. FAK is present in cells that form adhesion sites with the ECM or with other cells. It is also heavily involved in regulating angiogenesis and is considered a potential target for anti-angiogenic therapies in treating malignant tumors [150,151].

Markowska et al. investigated the roles of ANG1, CaSR, and FAK in uterine fibroids to explore their potential use in targeted therapies (Contribution 8). Their study found that ANG1 was expressed in both myoma and the surrounding tissue, as well as in the unaffected uterine tissue of women in the control group. However, among the 70 women in the study (with myoma and peripheral tissue) and the 12 women in the control group (with healthy uterine muscle), there were no significant differences in ANG1 expression levels (*p* = 0.983). In the experimental arm of the 70 women with uterine myomas, CaSR levels were measured in both the tumor tissue and its surrounding area. The concentrations of CaSR were found to be lower in the myoma and its periphery compared to the myometrium of healthy women (*p* = 0.001). These findings suggest that calcium supplementation may help prevent the growth of large myomas in women who already have small myomas. They also found that FAK expression is significantly reduced in uterine myomas and their surrounding tissue (18,338.54 ± 8824.04) compared to healthy uterine muscle (39,665.24 ± 14,231.92). This suggests that FAK does not play a role in the growth of benign tumors, making clinical trials of FAK inhibitors unnecessary.

Lung cancer is the second most prevalent cancer worldwide, and there is a growing need for diagnostic methods based on biomarkers that enable the early detection of lung cancer [152]. EVs from cancer cells modify the tumor microenvironment, facilitating the transformation of stromal cells into angiogenic, pre-metastatic, or tumor-suppressing cells [153,154]. Exosomes derived from cancer cells also display multiple tumor antigens on their surface, making them useful for the non-invasive early detection of cancer and monitoring treatment progress [155]. EVs play a crucial role in the diagnosis, treatment, and prognosis of non-small cell lung cancer by regulating the production of miRNAs, lncRNAs, circular RNAs (circRNAs), and proteins. Table 6 presents various components of extracellular vesicles (EVs) as biomarkers in NSCLC immunotherapy research.

Over the past decade, several methods have been developed to quantitatively evaluate exosomes, such as nanoparticle tracking analysis (NTA), Western blotting, fluorescence techniques, surface plasmon resonance (SPR), surface-enhanced Raman spectroscopy (SERS), and electrochemical sensing [168]. Of these, electrochemical detection stands out due to its simplicity, rapid response, high specificity, and accuracy [169]. Electrochemistry has diverse biomedical applications, and biosensors are analytical devices that use biological components to detect and quantify specific chemical compounds or biological molecules. Electrochemical biosensors utilize electrodes to monitor changes in electrical signals when a biological molecule attaches to a receptor on the electrode surface. Electrochemical techniques such as cyclic voltammetry and electrochemical impedance spectroscopy, known for their high sensitivity, user-friendliness, and minimal sample requirements, can be used to develop highly accurate biosensors [170]. Various materials, such as conductive polymers, metals, and semiconductors, can be employed to construct electrochemical biosensors [171]. To enhance the signal-to-noise ratio and sensitivity of electrochemical sensing systems, it is crucial to identify a material suitable for use as a surface that has properties like small size, a large electroactive surface area, good biocompatibility, and adequate conductivity.

Irani et al. sought to develop a reliable, reproducible, and sensitive biosensor for detecting and quantifying exosomes using electrochemical methods (Contribution 9). The process began with coating the electrode surface with a suitable layer of gold, followed by thermal annealing to create primary gold seeds. These seeds were then grown to the desired size, and the surface was activated by introducing functional groups with high-performance materials, carefully adhering to the required timing for each step. Subsequent steps involved designing system control tests to evaluate and confirm the biosensor’s functionality. To validate the biosensor, exosomes derived from cancer cells and human serum samples were used. Irani et al. concentrated on exosomes secreted by lung cancer cells (A549) and utilized the CD-151 antigen to differentiate these exosomes from other environmental factors. Their research employed multiple centrifugation steps at varying speeds, followed by an ultracentrifugation step at high speed to purify the exosomes. The custom-designed biosensor by Irani et al. achieved a limit of detection of 20 exosomes/mL in spiked blood serum, which is promising for further cancer screening applications. Electrochemical methods for detecting exosomes hold significant potential. Nevertheless, enhancing sensitivity, selectivity, and reproducibility, standardizing sample preparation protocols, and validating results across various platforms are challenges that should be addressed.

Glioblastoma (GBM) remains the most prevalent and aggressive glial tumor, with remarkable resistance to nearly all standard-of-care treatments, which typically include a combination of chemotherapy and radiation following surgical resection [172]. The difficulty in treating GBM primarily arises from a small population of therapy-resistant GBM stem cells (GSCs) and the complex inter- and intra-tumor heterogeneity, which encompasses various GBM subtypes and a diverse array of stromal cells within the TME [173,174,175]. GBM cells also recruit and alter immune cells distinct from microglia, fostering tumor growth and creating an immunosuppressive TME by releasing cytokines and EVs, and forming intercellular nanotubes [176]. Agosti et al. provide an in-depth review of emerging immunotherapeutic approaches targeting GBMs (Contribution 10). CAR-T cell therapy shines as a promising option, as these cells are meticulously engineered to target specific antigens. The advancement of second-generation CAR-T cells, designed for enhanced specificity and reduced off-target effects, underscores the potential of this therapy. Oncolytic viruses, which function through dual mechanisms, are currently being evaluated in clinical trials. Additionally, cancer vaccines, especially those aimed at neoantigens, present a personalized strategy with considerable potential. Finally, immune checkpoint inhibitors, such as PD-1 and cytotoxic T lymphocyte-associated protein 4 (CTLA-4) inhibitors, have shown promise in clinical trials.

Given the pivotal role of GSCs in glioma biology, there is growing interest in developing targeted therapies to eradicate these cells. Agosti et al. examined the molecular mechanisms underlying glioma progression linked to GSCs and pinpointed the critical signaling pathways and molecular interactions involved in GSC maintenance, resistance to therapy, and tumor recurrence (Contribution 11). Targeting the Notch, PI3K/AKT, and Wnt/β-catenin signaling pathways with specific inhibitors offers a promising approach to disrupting GSC maintenance and tumor growth. The advancement of these targeted therapies, along with sophisticated genomic and proteomic profiling, opens the door to personalized treatment strategies in glioma.

Prostatic adenocarcinoma is the second most commonly diagnosed cancer in men, with an estimated 1.4 million cases and 375,000 deaths worldwide in 2020 [177]. It serves as a classic example of an epithelial tumor that, in its well-differentiated stages, resembles normal prostate glands [178]. As the tumor advances, this normal-like architecture becomes increasingly disorganized, progressing to a poorly differentiated state. It is hypothesized that across all these tumor differentiation subtypes, the proliferation and growth of carcinomas are driven by a critical cellular compartment that resembles normal stem cells—these are often referred to as cancer stem cells (CSCs). Koukourakis et al. provided a comprehensive review of the theories concerning the origin of prostate cancer stem cells (PCSCs), their characterization, and the molecular pathways they engage (Contribution 12). PCSCs have been identified and partially characterized, displaying surface markers such as CD44, CD133, and integrin α2β1, as well as embryonic proteins like OCT4, NANOG, and SOX. These cells exhibit the activation of key signaling pathways, including Notch, NF-kB, PTEN/Akt/PI3K, RAS-RAF-MEK-ERK, and Hedgehog. The overexpression of stem cell markers and the hyperactivation of these pathways have been effectively employed to predict therapeutic responses and offer prognostic insights [179]. PCSCs are essential for replenishing the population of prostate cancer cells during radiotherapy and chemotherapy, and they exhibit notable resistance to androgen deprivation therapy [180]. Consequently, the incorporation of stem cell-targeting agents is expected to improve the effectiveness of these therapeutic approaches.

In summary, evaluated herein studies of the molecular biology of cancer with emerging biomarkers and therapeutic advances showcase the impressive progress along with drawbacks and challenges. Within the broader context of translational and clinical cancer research, these developments are anticipated to enable more sensitive, cost-effective, and non-invasive tests to facilitate timely and accurate diagnosis and personalized engagement, leading to prevention or precision-guided therapies. Unlike traditional anticancer treatments, innovative strategies such as triggered release mechanisms, intracellular drug targeting, cancer stem cell therapy, magnetic drug targeting, and ultrasound-mediated drug delivery, along with gene delivery, have led to the development of new treatment modalities for cancer. The crucial task in the development of anticancer drugs involves achieving site-specific delivery while minimizing systemic toxicity. Tumors create a dynamic environment where factors such as angiogenic potential, cell mass, and extracellular matrix composition constantly changing. Recent advances in drug delivery strategies are identifying preventive interventions, allowing new chemopreventive agents to be delivered via novel cell-targeting methods. Targeted drug delivery can be achieved by exploiting the overexpression of transporters and receptors on the plasma membrane of cancer cells. Additionally, ion channels such as potassium, sodium, calcium, chloride, and AQP4 channels may be targeted to regulate tumor metastasis. Cancer cells rely on these channels for migration. Overall, combined with promising immunotherapies and precision delivery systems, early detection and interventions with a customized approach is anticipated to improve the outcomes of existing treatments and enable new mono- and combined therapies, minimizing harmful side effects and improving the targeting of specific mechanisms with desirable outcomes. In this exciting journey guided by the molecular biology of cancer, it is hoped that the acquired knowledge summarized in this compendium will assist in advancing the frontiers of cancer therapy for better management and care.

## Figures and Tables

**Table 1:** An overview of 11 studies on the roles of miRNA-27a and miRNA-128 in breast cancer.

MicroRNA	Biofluid	Type Deregulation	Reference
miRNA-27a	Serum	↑ breast cancer vs. control	[16]
	Plasma	↑ breast cancer vs. control ↑ late breast cancer vs. early breast cancer = benign vs. control vs. high risk breast cancer = high risk breast cancer vs. control	[17]
	Plasma	↑ breast cancer vs. control	[18]
	Serum	↑ primary breast cancer vs. benign breast lesions ↑ primary breast cancer vs. control ↑ benign breast lesions vs. control	[19]
	Plasma	↑ breast cancer vs. control ↓ breast cancer after chemotherapy vs. breast cancer before chemotherapy ↑ breast cancer after chemotherapy vs. control	[20]
	Serum	↓ breast cancer vs. control	[21]
	Plasma	↓ breast cancer vs. control	[22]
	Serum	↑ breast cancer vs. control	[23]
	Plasma	↑ breast cancer vs. control	[24]
miRNA-128	Plasma	↓ breast cancer vs. high-risk breast cancer	[25]
	Serum	= breast cancer vs. control	[26]

**Table 2:** Diagnostic methods used for hereditary and somatic breast cancer.

Nucleic Acid-Based Molecular Diagnostics of the Breast Cancer			
	Hereditary cancer-linked gene mutations	Non-hereditary tumor genomic mutations	Tumor gene expression profile
Method	Next-generation sequencing	Next-generation sequencing	RNA expression assay
Applicable samples	Tissue (isolated DNA) Blood (isolated DNA from leukocytes)	Tumor tissue (isolated DNA) Blood (isolated circulating tumor or cell-free DNA)	Tumor tissue (isolated RNA)
Genes or number of genes tested	*BRCA1*, *BRCA2*, *P53*, *PTEN*, *CDH1*, *PALB2*, and other genes	From 2 to over 400	Depends on assay 7–80
Gained biological information	Germline DNA mutations, deletions, amplifications, and fusions	Mutations, deletions, amplifications, and fusions in tumor DNA	Alterations of gene expression in tumor tissue
Clinical relevance	Identification of patients for targeted therapy	Prognostic information, possible gene targets for targeted therapy and information about recurrence or resistance to treatment	Prognostic information and prediction of benefit from chemotherapy

**Table 3:** MiRNAs used as biomarkers for the early diagnosis of colorectal cancer.

MiRNA	Abundance	Target	Impact on Colorectal Cancer	Reference
miR-21	High	MEG2	Promoting cell proliferation and inhibit apoptosis	[54]
miR-485–3p	Low	TPX2	Inhibiting cell proliferation	[55]
miR-4728–5p	Low	MST4	Inhibiting cell proliferation	[55]
miR-3937	High	BCL2L12	Promoting cell invasion and migration	[56]
miR-31	High	RAS p21 GTPase	Promoting cell proliferation	[57]
miR-22–3p	Low	KDM3A	Inhibiting the proliferation, migration and invasion	[58]
miR-20a	High	Smad4, E-cadherin	Promoting the invasion and migration	[59]
miR-145	Low	Fasin-1, MYC	Inhibiting cell proliferation and metastasis	[59]
miR-223	High	FBXW7	Promoting proliferation, invasion and migration	[60,61]
miR-182	High	High MTDH	Enhancing colorectal cancer cell survival, invasion, and drug resistance	[61,62]
miR-92a	High	Bim	Promoting colorectal cancer cell proliferation, invasion and migration	[63]
miR-18a	Low	CDC42	Making cell cycle arrest, promote apoptosis	[64]

**Table 4:** The variability in CDX2 negative tumor assessment.

Detection Method	CDX2 (−) (%)	Patients (n) CDX2 (−)/Total	Independent DFS Predictor	Reference
mRNA	4.1	87/2115	NA	[71]
mRNA	6.9	32/466	yes	
protein	12.1	38/314	yes	
protein	5.3	8/150	yes	[72]
protein	5.9	42/713	yes	[73]
protein	10.9	25/232	in MSS CRC	[74]
protein	10.0	102/1003	no	[75]
protein	6.0	39/637	yes	[76]
mRNA	15.6	73/469	yes	[77]
protein	29.0	183/621	in familial CRC	[78]
protein	11.6	66/571	in combined cohort	[79]
	8.5	50/586		

Abbreviations: DFS, disease-free survival; mRNA, messenger RNA; MSS, microsatellite stable; CRC, colorectal cancer; NA, non-applicable.

**Table 5:** Selected clinical trials combining ICIs and CAR-T therapy.

Clinical Trials	Phase	Number of Patients	ICI	CAR-T Cell Target	Cancer Type	Preliminary Results
NCT01822652	I	11	Pembro	GD2	Neuroblastoma	6 PD, 2 CR (after salvage), 5 SD
NCT02414269	I	27	Pembro	Mesothelin	Malignant pleural diseases, comprising metastatic lung and breast cancers and malignant pleural mesothelioma	2 CR, 8 SD (>6 months)
NCT03287817	I	19	Pembro	CD19/22 dual target	r/r DLBCL	64% ORR, 55% CRR
NCT03630159	Ib	4	Pembro	CD19	r/r DLBCL	1 PR, 2 PD
NCT03726515	I	7	Pembro	EGFRvIII	EGFRvIII + , MGMT-unmethylated glioblastoma	Low efficacy, 7 PD, median PFS: 5.2 months, median OS: 11.8 months
NCT04991948	Ib	Estimated 34	Pembro	NKG2D	Colorectal cancer	2 deaths reported
NCT04995003	I	Estimated 25	Pembro or nivo	HER2	Advanced Sarcoma	NA
NCT04003649	I	Estimated 60	Nivo and ipi	IL13Ra2	Glioblastoma	NA
NCT04539444	II	16	Tisleli	CD19/22 dual target	R/R B-NHL	CR 11, 1-year PFS: 68.8%, 1-year OS: 81.3%
NCT04381741	Ib	8	Tisleli	CD19	R/R DLBCL	4 CR, 1 PR, 2 PD
NCT02926833	I/II	28	Atezo	CD19	DLBCL	75% ORR, 46% CR, 29% PR,7%SD, 14%PD
NCT02706405	I	29	Durva	CD19	R/R LBCL	35% ORR, 27% CR
NCT03310619	I/II	Estimated 77	Durva, nivo, Relatli	CD19	R/R aggressive B-cell NHL	NA

Abbreviations: ICI, immune checkpoint inhibitor; CAR-T cell, chimeric antigen receptor-T cell; Pembro, pembrolizumab; PD, progression disease; CR, complete response; SD, stable disease; R/R DLCBL, relapsed/refractory diffuse large B cell lymphoma; ORR, overall response rate; CRR, complete response rate; PR partial, response; EGFRvIII, epidermal growth factor receptor variant III; MGMT, O^6^-methylguanine-DNA methyltransferase; PFS, progression-free survival; OS, overall survival; NKG2D, natural killer group 2D; Nivo, nivolumab; HER2, epidermal growth factor receptor 2; NA, non-applicable; Ipi, ipilimumab; IL13Ra2, interleukin-13 receptor subunit alpha-2; Tisleli, tislelizumab; R/R B-NHL, relapsed/refractory B-cell non-Hodgkin lymphoma; R/R DLCBL, relapsed/refractory diffuse large B cell lymphoma; Atezo, atezolizumab; Durva, durvalumab; R/R LBCL, relapsed/refractory large B cell lymphoma; Relatli, relatlimab.

**Table 6:** Various components of EVs as biomarkers in NSCLC immunotherapy research.

Biomarkers	Expression	Recipient Cell	Target	Function	Reference
miR-21	Up	HBE cells	STAT3	Blocking angiogenesis	[156]
miR-494 miR-524-3p	Up	AD cells	LnStr, LFb	Regulating organs prior to tumor metastasis	[157]
LINC00301	Up	NSCLC cells	TGF-b	Impact on NSCLC development	[158]
lncRNA UFC1	Up	NSCLC cells	EZH2	Impact on NSCLC development	[159]
CircNDUFB2	Up	NSCLC cells	IGF2BPs	Stimulates anti-tumor immunity	[160]
has-circRNA-002178	Up	AD cells	None	Promoting PDL1/PD1 expression in lung AD	[161]
circUSP7	Up	NSCLC cells	CD8+ T cells	Promotion of immunosuppression	[162]
PKM2	Up	NSCLC cells	None	Promotes NSCLC cell proliferation and CDDP resistance	[163]
PLA2G10	Up	NSCLC cells	None	Negative correlation with NSCLC prognosis	[164]
GCC2	Up	NSCLC cells	None	Early diagnostic biomarkers for NSCLC	[165]
GCC2-ALK	Up	NSCLC cells	ALK	For NSCLC diagnosis and treatment	[166]
LBP	Up	NSCLC cells	None	Diagnosis of metastatic NSCLC	[167]

Abbreviations: EVs, extracellular vesicles; NSCLC, non-small cell lung cancer; AD, adenocarcinoma; CDDP, cisplatin.
